# Supplementary material for: Exploring the past and forecasting the future of malaria in selected Nigerian states: A time series modelling approach using wavelet and SARIMA
Source: PLoS One. 2026 Apr 2;21(4):e0337791. doi: 10.1371/journal.pone.0337791 (PMC13046133; doi:10.1371/journal.pone.0337791)
Supplement: Attachment — Submitted filename: Malaria Time_series Review Response1.docx [file pone.0337791.s001.docx]

**Response to Handling Editor’s Comments**

**Manuscript Title:** Exploring the past and forecasting the future of malaria in selected Nigerian states: A time series modelling approach using wavelet and SARIMA

**Submission ID:** PONE-D-25-59526

**Handling Editor’s Comments**

1. We note you write" The data underlying the results presented in the study are available from (National Malaria Data Repository, https://nmdrnigeria.ng/dhis-webcommons/security/login.action#/vfXbRtibw3W)." however, this requires a login.

**Authors’ response**

We thank the handling Editor for this comment. All relevant data for this study are publicly available from the figshare repository (https://doi.org/10.6084/m9.figshare.30716729)

2. Please also confirm that none of the authors had access to identifying information when accessing the data.

**Authors’ response**

None of the authors had access to any identifying information at any stage of the study. All data were provided in fully de-identified form.

3. Overall, please add in your method section of your manuscript:

A.) the source of the data (link)

B.) dates when you accessed the data

C.) that data was accessed in de-identified manner

**Authors’ response**

We thank the handling Editor for this comment. These have all been addressed in the manuscript (See page 4, line 125 to 131).
