# Supplementary material for: Exploring the past and forecasting the future of malaria in selected Nigerian states: A time series modelling approach using wavelet and SARIMA
Source: PLoS One. 2026 Apr 2;21(4):e0337791. doi: 10.1371/journal.pone.0337791 (PMC13046133; doi:10.1371/journal.pone.0337791)
Supplement: Attachment — Submitted filename: Response to Reviewers (1).docx [file pone.0337791.s002.docx]

**Response to Reviewers’ Comments**

**Manuscript Title:** Exploring the past and forecasting the future of malaria in selected Nigerian states: A time series modelling approach using wavelet and SARIMA

**Submission ID:** PONE-D-25-59526

**Editor’s Comments**

1. Please ensure that your manuscript meets PLOS ONE's style requirements, including those for file naming. The PLOS ONE style templates can be found at[https://journals.plos.org/plosone/s/file?id=wjVg/PLOSOne_formatting_sample_main_body.pdf](https://journals.plos.org/plosone/s/file?id=wjVg/PLOSOne_formatting_sample_main_body.pdf) and [https://journals.plos.org/plosone/s/file?id=ba62/PLOSOne_formatting_sample_title_authors_affiliations.pdf](https://track.editorialmanager.com/CL0/https://journals.plos.org/plosone/s/file?id=ba62/PLOSOne_formatting_sample_title_authors_affiliations.pdf/1/010f019b6a2bbcc6-6eadaa1c-e5b5-4cf0-9adf-67b046424fa0-000000/01ynimjc6dqlq4DYggSonItJfK71mKypr4l77YHV10M=243)

**Authors’ response**

We thank the Editor for this comment. We have ensured that our manuscript meets PLOS ONE’s style.

2. Please note that PLOS One has specific guidelines on code sharing for submissions in which author-generated code underpins the findings in the manuscript. In these cases, we expect all author-generated code to be made available without restrictions upon publication of the work. Please review our guidelines at [https://journals.plos.org/plosone/s/materials-and-software-sharing#loc-sharing-code](https://journals.plos.org/plosone/s/materials-and-software-sharing#loc-sharing-code) and ensure that your code is shared in a way that follows best practice and facilitates reproducibility and reuse

**Authors’ response**

We thank the Editor for this comment. The author-generated code has been made available on <https://github.com/haryodolapo/Malaria-Forecasting-Nigeria>

3. We note that the grant information you provided in the ‘Funding Information’ and ‘Financial Disclosure’ sections do not match. When you resubmit, please ensure that you provide the correct grant numbers for the awards you received for your study in the ‘Funding Information’ section.

**Authors’ response**

We thank the Editor for this observation. This has been addressed in the submission platform.

4. Thank you for stating the following financial disclosure: INV-047051- Bill and Melinda Gates Foundation Grants on Capacity development in Malaria and NTDs Data Modelling.
   At this time, please address the following queries:

**Authors’ response**

We thank the Editor for this observation. This has been clarified in the cover letter.

**5** We note that you have indicated that there are restrictions to data sharing for this study. For studies involving human research participant data or other sensitive data, we encourage authors to share de-identified or anonymized data. However, when data cannot be publicly shared for ethical reasons, we allow authors to make their data sets available upon request. For information on unacceptable data access restrictions, please see [http://journals.plos.org/plosone/s/data-availability#loc-unacceptable-data-access-restrictions](http://journals.plos.org/plosone/s/data-availability#loc-unacceptable-data-access-restrictions).

**Authors’ response**

We thank the Editor for this observation. We have uploaded an anonymized dataset in a repository. All relevant data for this study are now publicly available from the figshare repository <https://doi.org/10.6084/m9.figshare.30716729>

6. When completing the data availability statement of the submission form, you indicated that you will make your data available on acceptance. We strongly recommend all authors decide on a data sharing plan before acceptance, as the process can be lengthy and hold up publication timelines. Please note that, though access restrictions are acceptable now, your entire data will need to be made freely accessible if your manuscript is accepted for publication. This policy applies to all data except where public deposition would breach compliance with the protocol approved by your research ethics board. If you are unable to adhere to our open data policy, please kindly revise your statement to explain your reasoning and we will seek the editor's input on an exemption. Please be assured that, once you have provided your new statement, the assessment of your exemption will not hold up the peer review process.

**Authors’ response**

We thank the Editor for this observation. We have now revised our statement in the submission platform.

7. We note that Figure 1 in your submission contain map image which may be copyrighted. All PLOS content is published under the Creative Commons Attribution License (CC BY 4.0), which means that the manuscript, images, and Supporting Information files will be freely available online, and any third party is permitted to access, download, copy, distribute, and use these materials in any way, even commercially, with proper attribution. For these reasons, we cannot publish previously copyrighted maps or satellite images created using proprietary data, such as Google software (Google Maps, Street View, and Earth). For more information, see our copyright guidelines: [http://journals.plos.org/plosone/s/licenses-and-copyright](http://journals.plos.org/plosone/s/licenses-and-copyright).

**Authors’ response**

We thank the Editor for this observation. This has been addressed in the revised manuscript. (See the highlighted part of the caption in Fig. 1 on page 4)

**Reviewer 1 Comments**

1. More details on the application of wavelet analysis to investigate malaria, along with the gaps and or the limitations in those studies

**Authors’ response**

We thank the Reviewer for this comment. We have expanded the literature review to include more studies that applied wavelet analysis in investigating malaria along with the notable gap in these studies. See the highlighted part on page 3, lines 75-89.

2. As we are approaching the end of 2025, it would be valuable to compare the forecasted values with the observed incidence data from 2025.

**Authors’ response**

We thank the Reviewer for this observation. We do not currently have access to the updated and high-quality data for 2025. We however used the incidence data from January 2023 to December 2024 as test data to compare with the forecasted values for the same period of time. We selected the models with the best predictive accuracy. (See page 13, lines 432-445 and Fig. 7).

**Reviewer 2 Comments**

1. First, the description of data preprocessing is insufficient. Given the use of routine surveillance data, the authors should clearly explain how missing values, reporting artefacts, and outliers were handled to ensure reproducibility. In addition, the analyses are based on absolute case counts without population adjustment, which may bias comparisons across states with different population sizes and growth trends. The authors should justify this choice or consider sensitivity analyses using incidence rates.

**Authors’ response**

We thank the Reviewer for this comment. Data preprocessing and population adjustment have now been addressed in the revised manuscript (See page 4, line 126 and page 5, lines 140-152).

2. For the wavelet analysis, the manuscript would benefit from clearer reporting of the statistical significance framework, including how significance levels were defined and tested. Some periodic features are interpreted despite falling largely within the cone of influence, and these results should be treated more cautiously. Interpretation of bivariate wavelet synchrony should also acknowledge the possibility that shared seasonality or common external drivers (e.g. rainfall patterns or intervention timing) may explain observed coherence, rather than direct epidemiological coupling.

**Authors’ response**

We thank the Reviewer for these comments. The statistical significance framework has been reported clearer in the revised manuscript (See page 9, lines 315-333). We have emphasized that features that fall within the cone of influence should be interpreted with caution (See page 10, lines 352-353, lines 361-362; page 12, lines 400-401 and lines 408-409). We acknowledged that synchrony between states are likely due to shared seasonal patterns and climatic drivers (See page 15, lines 501-503).

3. Model selection is automated, but diagnostic results are only briefly described. Residual plots and Ljung–Box results should be more clearly reported or summarized to support model adequacy. Forecasts are presented clearly, but interpretation focuses heavily on point estimates. Greater emphasis on prediction intervals and uncertainty would strengthen the public health relevance.

**Authors’ response**

We thank the Reviewer for these comments. Diagnostic results have now been described in detail (See page 13, lines 446-460). We have now presented prediction intervals in the revised manuscript (See the part highlighted red in the Abstract and on page 16, lines 523-539).

4. The study period includes major programmatic and contextual changes (e.g., SMC scale-up, COVID-19). These potential structural breaks are not formally assessed and should be discussed as a limitation. Although the data are aggregated and de-identified, the ethics statement is listed as “N/A.” Clarification on ethical approval or waiver for secondary use of routine health data is recommended.

**Authors’ response**

We thank the Reviewer for these comments. This has been addressed in the revised manuscript (See page 16, lines 549-551).

5. Regarding the SARIMA models, automated model selection is acceptable, but diagnostic results are only briefly summarized. More explicit presentation or description of residual diagnostics would strengthen confidence in the models. Forecast results should place greater emphasis on uncertainty and prediction intervals, particularly given the variability of malaria surveillance data.

**Authors’ response**

We thank the Reviewer for these comments. Diagnostic results have now been described in detail (See page 13, lines 446-460). We have now presented prediction intervals in the revised manuscript (See the part highlighted red in the Abstract and on page 16, lines 523-539).

6. Figures are generally informative but would benefit from clearer labeling. Figure captions should more clearly explain the cone of influence and statistical significance. Forecast figures would be improved by clearer distinction between observed and predicted values and by emphasizing prediction intervals. Tables are mostly clear, but additional explanation of abbreviations and model terms in footnotes would improve readability.

**Authors’ response**

We thank the Reviewer for these comments. These observations have now been addressed in the revised manuscript (See the captions of Fig 3 and Fig 4 on pages 10 and 11 respectively. Also see Fig 7 and Fig 8 for the improved labeling). Footnotes have been added to explain the abbreviations used in Table 3 (See page 14).

7. The manuscript is generally intelligible but requires substantial language editing. There are frequent grammatical and stylistic issues, along with some repetition, especially in the Introduction and Discussion sections. Improving clarity and conciseness would significantly enhance readability.

**Authors’ response**

We thank the Reviewer for these comments.We have carefully revised the manuscript, especially the Introduction and Discussion sections to improve clarity, conciseness, and grammatical accuracy.
