# Supplementary material for: Exploring the past and forecasting the future of malaria in selected Nigerian states: A time series modelling approach using wavelet and SARIMA
Source: PLoS One. 2026 Apr 2;21(4):e0337791. doi: 10.1371/journal.pone.0337791 (PMC13046133; doi:10.1371/journal.pone.0337791)
Supplement: Attachment — Submitted filename: Response to Reviewers (2).docx [file pone.0337791.s003.docx]

**Response to Reviewers’ Comments**

**Manuscript Title:** Exploring the past and forecasting the future of malaria in selected Nigerian states: A time series modelling approach using wavelet and SARIMA

**Submission ID:** PONE-D-25-59526

**Editor’s Comments**

1. Clarification on Data Preprocessing for SARIMA:
   The manuscript states that for the SARIMA model, the time series was log-transformed to stabilize variance. However, it is unclear if absolute case counts or incidence rates were used as the input for this log transformation. This must be explicitly stated in the “Data description and preprocessing” section. For consistency and to avoid confounding by population size in the forecasts, please clarify and justify the choice of using case counts versus rates for the SARIMA modeling.

**Authors’ response**

We thank the editor for this insightful comment. We have updated the 'Data description and preprocessing' section (page 5, lines 144–146) to explicitly state that absolute case counts were used for the SARIMA models.

The rationale for this choice is based on the distinct objectives of our analyses. For the wavelet analysis, incidence rates were necessary to standardize the data across states, allowing for unbiased cross-wavelet comparisons and phase-relationship assessments without confounding by population size. In contrast, the SARIMA models were developed for individual state-level forecasting to assist in local resource planning. Since these models evaluate the temporal dependencies within each state's own history, absolute counts preserve the original scale and operational utility of the data. Furthermore, given that the underlying population remained stable over the study period, the use of counts does not introduce significant demographic confounding into the forecasts.

2. Ethics Statement Justification:
   The authors' response states, "The data used were secondary data and were obtained from National Malaria Data Repository. Hence, no need for ethical approval." While secondary analysis of anonymized aggregate data often qualifies for an exemption, PLOS ONE requires a formal statement to that effect.
   Please replace “N/A” in the Ethics Statement with a sentence such as: “This study involved the secondary analysis of fully anonymized, aggregate surveillance data obtained from the Nigerian National Malaria Data Repository. Ethical review and approval were waived for this study as it did not involve direct interaction with human subjects or access to identifiable personal information.

**Authors’ response**

We thank the editor for this observation. We have replaced “N/A” with the correct statement.

3. Validation of Forecasts:
   Reviewer 1's request to compare forecasts with 2025 data was reasonably addressed by using 2023-2024 as a test set. However, the text on page 13, lines 432-445 (and Fig. 7) should be made slightly more precise.
   Please modify the phrasing from “We selected the models with the best predictive accuracy” to “The model with the lowest error metrics on this test set was selected as the final model for each state.” This clarifies the model selection process post-testing.

**Authors’ response**

We thank the editor for this insightful comment. The texts on page 13 has now been re-written and has been made more precise (See page 13, lines 433-443 and Fig. 7 with its caption). We have also modified the phrase “best predictive accuracy” to “lowest error metrics” in the revised manuscript (See page 13, lines 435, 438 and 440).

4. Final Language and Consistency Check:
   While the language has improved, a thorough final proofread is necessary to catch minor grammatical errors and ensure stylistic consistency (e.g., consistent use of “wavelet” vs. “wavelets,” proper hyphenation in “time-frequency”).
   Please ensure all in-text citations and reference list entries match perfectly and are formatted according to PLOS ONE style.

We have conducted a thorough final proofread of the manuscript to ensure grammatical accuracy and stylistic consistency. Specifically, we have standardized the terminology to use “wavelet” as a descriptor (e.g., “wavelet analysis,” “wavelet transform”) and “wavelets” for the plural form of wavelet (e.g., family of wavelets, daughter wavelets). We have also ensured that “time-frequency” is consistently hyphenated throughout the text.
